# Adaptive restraint design for a diverse population through machine learning

**DOI:** 10.3389/fpubh.2023.1202970

**Published:** 2023-08-10

**Authors:** Wenbo Sun, Jiacheng Liu, Jingwen Hu, Judy Jin, Kevin Siasoco, Rongrong Zhou, Robert Mccoy

**Affiliations:** ^1^University of Michigan Transportation Research Institute (UMTRI), College of Engineering, University of Michigan, Ann Arbor, MI, United States; ^2^Department of Industrial and Operations Engineering, University of Michigan, Ann Arbor, MI, United States; ^3^Ford Motor Company, Dearborn, MI, United States

**Keywords:** adaptive design, machine learning, safety balance, Gaussian process, optimization

## Abstract

**Objective:**

Using population-based simulations and machine-learning algorithms to develop an adaptive restraint system that accounts for occupant anthropometry variations to further enhance safety balance throughout the whole population.

**Methods:**

Two thousand MADYMO full frontal impact crash simulations at 35 mph using two validated vehicle/restraint models representing a sedan and an SUV along with a parametric occupant model were conducted based on the maximal projection design of experiments, which considers varying occupant covariates (sex, stature, and body mass index) and vehicle restraint design variables (three for airbag, three for safety belt, and one for knee bolster). A Gaussian-process-based surrogate model was trained to rapidly predict occupant injury risks and the associated uncertainties. An optimization framework was formulated to seek the optimal adaptive restraint design policy that minimizes the population injury risk across a wide range of occupant sizes and shapes while maintaining a low difference in injury risks among different occupant subgroups. The effectiveness of the proposed method was tested by comparing the population-wise injury risks under the adaptive design policy and the traditional state-of-the-art design.

**Results:**

Compared to the traditional state-of-the-art design for midsize males, the optimal design policy shows the potential to further reduce the joint injury risk (combining head, chest, and lower extremity injury risks) among the whole population in the sedan and SUV models. Specifically, the two subgroups of vulnerable occupants including tall obese males and short obese females had higher reductions in injury risks.

**Conclusions:**

This study lays out a method to adaptively adjust vehicle restraint systems to improve safety balance. This is the first study where population-based crash simulations and machine-learning methods are used to optimize adaptive restraint designs for a diverse population. Nevertheless, this study shows the high injury risks associated with obese and female occupants, which can be mitigated via restraint adaptability.

## Introduction

Motor-vehicle crashes continue to be a public health problem in the United States. Field data analysis has shown an estimated 20, 160 people died in motor-vehicle crashes in the United States in the first half of 2021, up 18.4% over 2020, according to a report from the National Highway Traffic Safety Administration (1). Advanced technologies for restraint system designs have been widely explored to mitigate crash injuries. While current designs are effective in protecting people in a wide variety of frontal crash conditions, occupants with other body sizes can experience higher injury risks in crashes, severely hindering safety balance among the whole population (2, 3). Conceptually, a natural solution is to develop an adaptive restraint system that optimally adjusts the design settings to match the characteristics of different occupants and crash configurations. This study's focus is on the adaptivity regarding the occupant characteristics.

Some attempts have been made to develop adaptive restraint systems in the existing literature. For example, McCarthy et al. (4) and Reßle et al. (5) demonstrated the potential benefits of applying an adaptive restraint system to mitigate crash injuries based on field data. Shin et al. (6) and Miller and Maripudi (7) investigated the crash injuries under a combination of different restraint designs and several pre-determined crash conditions using simulations (8, 9). Untaroiu and Adam (10) searched for an optimal restraint law for each pre-crash posture classified from vision signals. Huang et al. (11) optimized the restraint design for five different sizes of occupants and two crash severities. Boyle et al. (12) optimized a restraint system for four occupants with a wide range of sizes and shapes under two pre-crash postures. However, all the existing studies were established based on pre-specified representation of occupants (for example, midsize male or 5th percentile female), which does not cover the heterogeneity of the whole population and may not protect occupants whose sizes are not represented by the pre-specified categories.

To achieve the population-wise protection, one natural way is to adjust an adaptive restraint system according to a pre-optimized design function that takes the occupant covariates [e.g., sex, stature, and body mass index (BMI)] as inputs and returns the optimal design setting. Such a function is called an “adaptive restraint design policy” in this paper and will be referred to as the “design policy” in the following context with no ambiguity. With the rapid development of vehicle technologies, especially with automated driving systems (ADS), the occupant covariates could be measured or estimated from available in-vehicle sensing signals, while the restraint system can be adaptively adjusted via an automatic control system. In this study, an optimal design policy is researched via machine learning generated from a large number of MADYMO occupant simulations. In the literature, a considerable number of machine learning methods have been proposed for design policies in the field of precision health. For example, Song et al. (13) utilized the Q-learning method to optimize a sequence of treatments based on patients' information. The O-learning approach was proposed by Zhao et al. (14) to formulate the adaptive design searching procedure as a classification problem for categorical decisions. In Gu et al. (15), a Bayesian model was developed to estimate the model parameters in the adaptive design function. However, those methods are not readily applicable to the problem where the inputs of the design policy are a mixture of continuous and categorical variables representing occupant size and shape.

In this study, a two-step procedure is proposed to learn the optimal design policy from the simulation dataset—(i) train a surrogate model to predict the injury risk for any given combination of occupant covariates and vehicle design variables; (ii) formulate and solve an optimization problem to seek an optimal design policy that can automatically assign restraint system settings adaptive to any given set of occupant covariates. To the authors' knowledge, this is the first study that explores the machine-learning algorithm for the optimal design of adaptive restraint systems to enhance the balance of crash protection. While solving this data-driven optimization problem, a machine learning methodology to search for the optimal design policy in the multi-dimensional space of continuous and categorical variables was delivered.

## Method

###  Notations and problem formulation

Throughout the paper, scalars are denoted by lowercase letters, vectors by lowercase boldface letters, and matrices by uppercase letters. Let d∈D denote the vehicle design variables and s∈S denote the occupant covariates. Here D and S represent the space of vehicle design variables and occupant covariates, respectively. The computer simulation model *f* is a function that takes ***d*** and ***s*** as inputs, and returns the injury risk as *f*(***s***, ***d***). Different from the traditional state-of-the-art designs, an adaptive restraint system adjusts the vehicle design variables ***d*** as a function of the occupant covariates ***s***, which is implemented via a design policy ***d*** = ***a***(***s***). The objective is to search for an optimal design policy that reduces the injury risk for the whole population characterized by the space of occupant covariates, S. Mathematically, the optimized target design policy is the solution to the following optimization problem:


(1) $$\begin{array}{l} a^{*}=\underset{a\in A}{arg min}{𝔼}_{s}\left[f\left(s,a\left(s\right)\right)\right]\\ \;=\underset{a\in A}{arg min}\underset{s\in S}{\int }f\left(s,a\left(s\right)\right)p\left(s\right)ds, \end{array}$$

where 𝔼_***s***_ is the expectation operator with respect to ***s***, *p*(***s***) is the probability density function of ***s*** for the occupant population, and A represents the space of all the feasible design policies.

**Figure 1:**
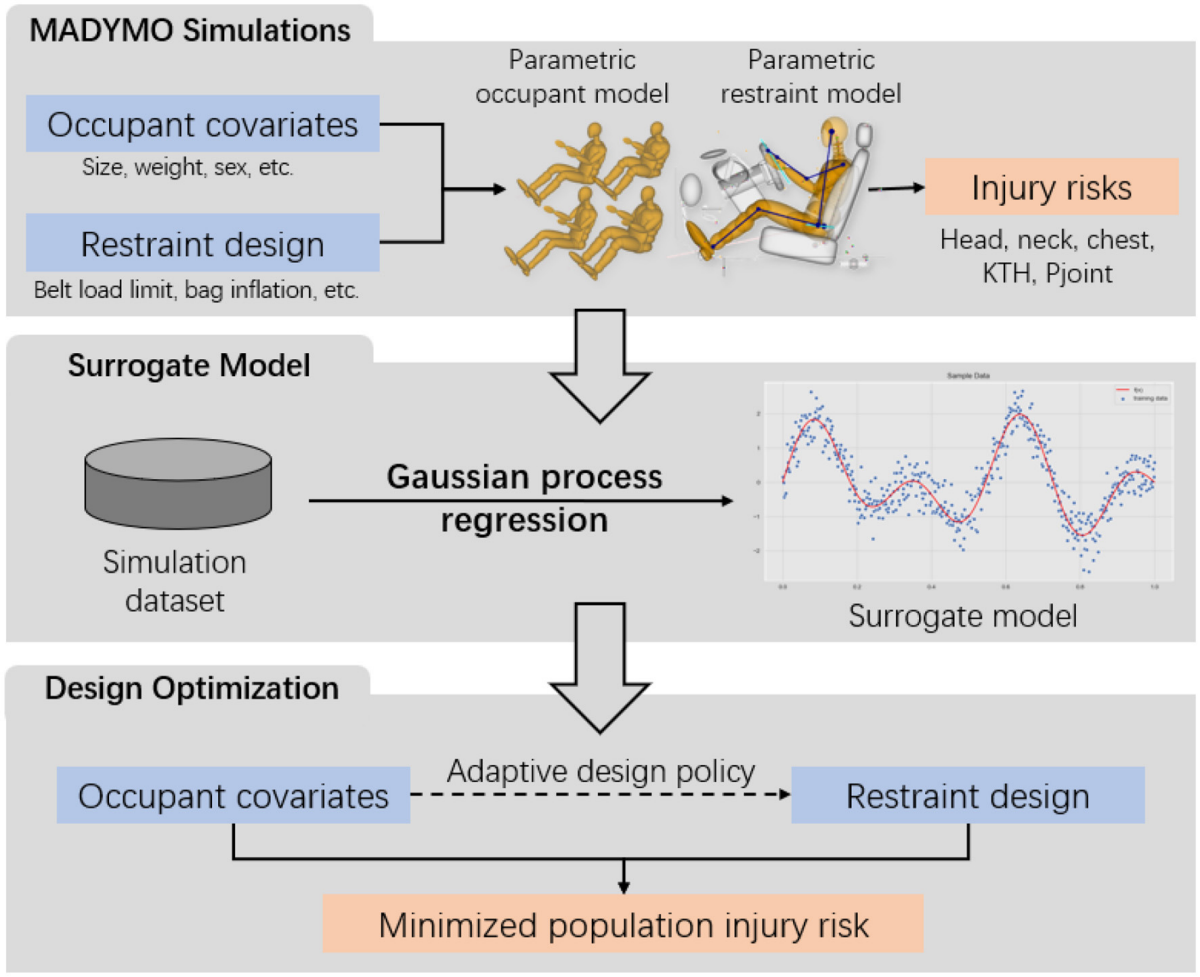
Flowchart of the proposed method. **(Upper panel)** The simulation data is generated from a MADYMO simulation model. **(Middle panel)** The simulation data is used to train a surrogate model. **(Lower panel)** The adaptive design policy is optimized to minimize the population injury risk. The bold texts highlight the key modules in the Method Section.

The optimization problem in Eq. (1) brings about three main research challenges. (i) Solving Eq. (1) requires the values of *f*(***s***, ***a***(***s***)) at different combinations of occupant covariates s∈S and vehicle design variables d∈D. Thus, it is necessary to construct a computer simulation model that can take any feasible vehicle design variables and occupant covariates as inputs to evaluate the corresponding injury risks. (ii) Although the injury risks can be evaluated from the computer simulation model, it is infeasible to generate all simulation runs at every point in the full space S×D under limited computational resources. A surrogate model $\hat{f}$, which is a computationally efficient prediction model for *f*, could be trained to directly generate fast predictions of the injury risk responses *f* given any inputs without running computational simulations. (iii) The injury risk function *f* is highly non-linear and non-convex, making the closed-form solution to Eq. (1) intractable. An efficient and accurate numerical solution scheme could be developed when searching for the optimal policy ***a***. The flowchart of the proposed method is depicted in Figure 1. Consequently, technical details to address the aforementioned research challenges will be presented.

###  Computer simulation model setup

In this study, two generic driver compartment models representing a midsize sedan and a midsize SUV were obtained from Ford Motor Company. Each of the two models includes a driver seat, safety belt, driver airbag, knee airbag, instrument panel, steering wheel, knee bolster, and other vehicle interior components. Both models have been rigorously validated against 35 mph full frontal impact crash tests with the Hybrid-III midsize male and small female dummies. A rigid-body-based scalable MADYMO dummy model was used to conduct all crash simulations. This parametric occupant model can represent both male and female occupants with a wide range of statures and body weights by scaling the midsize male occupant model using MADYMO scaler. The scaling is based on the anthropometric data of adults in the GEBOD (GEnerator of BODY data) database (16). In this study, the occupants were sampled within the 5th and 95th percentile stature and BMI in the male and female populations based on anthropometry data from the National Health and Nutrition Examination Survey (NHANES) for the years 2011-2014.

For each crash simulation, the scaled occupant model was positioned as a driver according to a driving posture model developed based on measurements from 68 volunteers (17). The driving posture model predicts occupant posture and position variables as a function of occupant body dimensions and vehicle package factors. In this study, only stature and BMI were used as the input parameters to define the driver sizes. The vehicle package factors, including seat height (H30), steering wheel X (distance from the center of steering wheel to the ball of foot reference point) (L6), and seat track angle (A27), were all configured based on the sedan and SUV model for predicting the driving postures. The model-predicted driver hip and eye locations (only in the X direction) were used to position the scaled occupant models, and the predicted driver-selected seat H-point location was used to position the seat before each simulation. For each simulation, the drivers hands were positioned on the steering wheel by adjusting the shoulder and elbow angles, and the right and left feet/shoes were positioned onto the gas pedal and the floor respectively by adjusting the hip, knee, and ankle angles. After the scaled occupant models and the vehicle seat were repositioned, the seat belt was fitted onto the occupant through pre-simulations.

To enable a large-scale simulation automation, ModeFrontier (ESTECO, Italy), a multi- disciplinary design optimization platform, MADYMO, and Scilab were used to integrate the occupant model scaling, occupant model positioning procedure, and automatic belt fitting algorithm together. Simulation runs were further used to train the surrogate model similar to that of Hu et al. (18). Generating more simulation runs can improve the accuracy of the surrogate model (19), while also bringing a higher computational cost. To balance the simulation time and model accuracy, 2,000 simulation runs were generated to explore the injury measures and the corresponding injury risks under a total of 10 occupant covariates and vehicle design variables for each vehicle type, sedan and SUV included. The vehicle design variables and the associated ranges are in Table 1. Three main injury measures were collected from the simulation model: head injury criterion (HIC = HIC15), chest deflection (ChestD), and femur forces (FF). Following the techniques introduced in Hu et al. (20), a regression model was trained to estimate the chest depth and femur cross-sectional area based on occupant covariates. The chest and lower extremity injury risks of each occupant were estimated via scaling the injury risk curve of a midsize male to the corresponding size. Finally, the joint injury risk function (Pjoint) was evaluated by integrating the injury risks at different body regions.

**Table 1:** Summary of vehicle design variables.

**Name**	**Explanation (unit)**	**Min**	**Max**
D0_LL	Load limiter belt payout D0 (m)	0.03	0.05
D1_LL	Load limiter belt payout D1 (m)	0.01	0.11
DAB	Airbag cushion diameter (m)	0.96	1.12
DABSTRAP	Airbag tether (m)	0.2032	0.3048
F_LL	Retractor belt force (N)	1,000	4,000
MF	Adjusted percentage from the default airbag mass flow rate	−15%	+15%
Stiff	Adjusted percentage from the default knee bolster stiffness	−40%	+40%

To effectively utilize the information from simulation runs, the simulation inputs were determined by the state-of-art design of experiments technique “Maximum Projection Design” (21). Consequently, for each vehicle type, the simulation runs are collected in a simulation dataset of *n* = 2, 000 triplets (***s***_*i*_, ***d***_*i*_, ***y***_*i*_; *i* = 1, ..., *n*) where ***s***_*i*_, ***d***_*i*_ and ***y***_*i*_ represent the occupant covariates, vehicle design variables, and injury measures in the *i*-th simulation run, respectively.

###  Surrogate model training

In this subsection, a surrogate model is trained to predict injury measures ***y***_*i*_ based on the input ***s***_*i*_ and ***d***_*i*_. The predicted injury measures can be directly used to evaluate the injury risks (20). Among the variety of choices of the surrogate model, the Gaussian Process (GP) modeling (22) was utilized because it not only provides a point estimation but also quantifies the prediction uncertainty. The uncertainty quantification of the surrogate model grants us the possibility to evaluate the potential worst-case scenario when predicting the injury risks and allows us to enhance the robustness of the target design policy.

In the GP modeling, the MADYMO simulation outputs of injury measures were considered as a random function that follows a GP. The GP surrogate model directly predicts the MADYMO simulation output of injury measures at any given input point as a weighted summation of *n* initial simulation outputs of {***y***_*i*_; *i* = 1, ..., *n*}, wherein the weights are determined based on this input point's relative distances to those simulated input points of {(***s***_*i*_, ***d***_*i*_; *i* = 1, ..., *n*)}. The GP is uniquely determined by a mean function and a covariance function, which are parameterized by hyper-parameters to be estimated via Maximum Likelihood Estimation (MLE). Further mathematical details can be found in Rasmussen (22). In this study, the GP-based surrogate model is implemented by the Python package “GPyTorch” (23) with the Squared Exponential Kernel function. To ensure the robustness of the resultant design policy in the worst-case scenarios, the 95% upper confidence bound from “GPyTorch” was selected as the prediction of the injury measures. The predicted injury measures are then mapped into the injury risks according to Hu et al. (20). After training the GP surrogate model, the GP model can be used to directly predict the injury risks and the Pjoint, $\hat{f}\left(s,d\right)$, given any pair of the simulation input variables (***s***, ***d***) without conducting MADYMO simulations.

###  Design policy optimization

In Eq. (1), *f* is substituted with the trained surrogate model $\hat{f}$, and the optimization problem is rewritten as


(2) $$\begin{array}{l} â=\underset{a\in A}{arg min}\underset{s\in S}{\int }\hat{f}\left(s,a\left(s\right)\right)p\left(s\right)ds. \end{array}$$

For the dimension reduction purpose, ***a***(***s***) is expressed in a polynomial form as


(3) $$\begin{array}{l} a\left(s\right)=\overset{m}{\underset{j=1}{\sum }}B_{j}\phi_{j}\left(s\right), \end{array}$$

where *B*_*j*_ is the matrix of coefficients for the *j*-th basis, $\phi_{j}\left(s\right)=\left[{s_{1}}^{j-1},\ldots ,s_{K}^{j-1}\right]$, and *s*_*k*_ denoting the *k*-th dimension of ***S*** with the maximal dimension *K*. Let *B* = {*B*_*j*_; *j* = 1, ..., *m*}. The optimization problem in Eq. (2) is converted into


(4) $$\begin{array}{l} \hat{B}=\underset{B\in B}{arg min}\underset{s\in S}{\int }\hat{f}\left(s,\overset{m}{\underset{j=1}{\sum }}B_{j}\phi_{j}\left(s\right)\right)p\left(s\right)ds. \end{array}$$

For ease of computation, the integral in Eq. (4) is evaluated through Monte Carlo simulation, that is, to first sample a subset of occupant covariates S⊂S according to the probability density function *p*(***s***), and then solve


(5) $$\begin{array}{l} \hat{B}=\underset{B\in B}{arg min}\underset{s\in S}{\sum }\hat{f}\left(s,\overset{m}{\underset{j=1}{\sum }}B_{j}\phi_{j}\left(s\right)\right). \end{array}$$

Now the optimization function in Eq. (5) is directly calculable for any given *B*. Due to the non-convexity of the optimization function, we adopt the non-convex optimization problem solver, Adam (24), with the autograd function (25) in Python to solve out the coefficients in the design policy, $\hat{B}$.

Substituting $\hat{B}=\left\{{\hat{B}}_{j};j=1,...,m\right\}$ into Eq. (3) yields the resultant design policy:


(6) $$\begin{array}{l} \hat{a}\left(s\right)=\overset{m}{\underset{j=1}{\sum }}{\hat{B}}_{j}\phi_{j}\left(s\right), \end{array}$$

which adaptively returns the vehicle design variables as a function of the occupant covariates ***s***.

## Results

**Figure 2:**
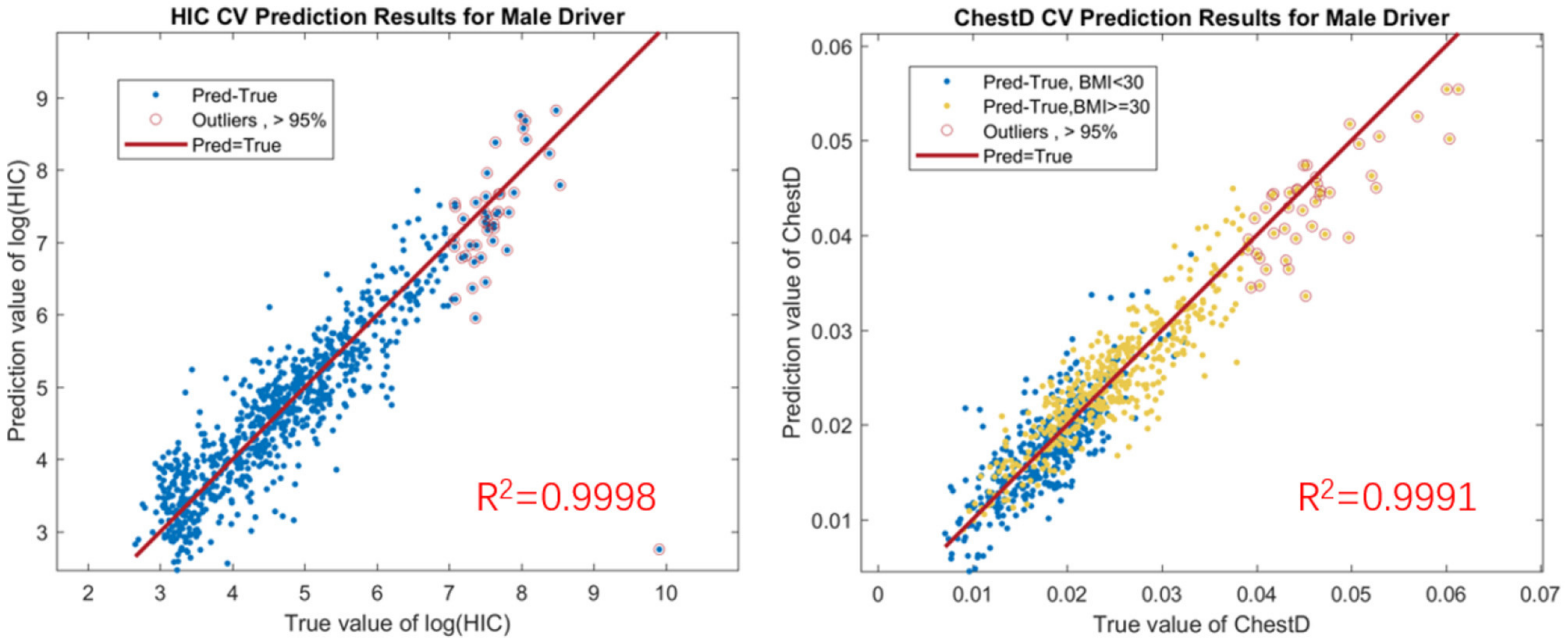
**(Left panel)** 10-fold cross validation of GP predictions for HIC values on male drivers in sedans. **(Right panel)** 10-fold cross validation of GP predictions for chest deflection values on male drivers in sedans. HIC is presented in a logarithmic transformation to better visualize the correlation between predicted and true values in a reasonably small scale.

A total of 2, 000 MADYMO simulations were conducted for the sedan and SUV model. The design of experiment matrices were randomly generated based on the Maximum Projection Design (21), which shows advantages in the accuracy of the trained surrogate model among a variety of applications. The surrogate model was trained via the Gaussian process regression technique, where the squared exponential kernel was selected. The accuracy of the surrogate model was evaluated based on the prediction errors in a 10-fold cross-validation setup. Simulation runs were divided into 10 folds. The surrogate model was trained based on the data from nine folds, which was used to generate the predictions at the left-out fold. The procedure was repeated for 10 times for each fold. The prediction errors are then visualized in a scatter plot of the predicted and true values among all the test samples in all the 10 folds. As an illustration, the cross-validation results for HIC values and chest deflection values on male drivers for the sedan model were shown in Figure 2, where most of the points lie on the line of *y* = *x*, indicating the high accuracy of the surrogate model. The occupants whose BMI is greater than the 95% population quantile is depicted by the circles. Although these points show a higher variance than those for subjects with a normal BMI, the predicted values are still close to the true values. The *R*^2^ value of the predictions on the left and right panels are 0.9998 and 0.9991, respectively. The comparison results also suggest that it is sufficient to generate 2, 000 simulations for each vehicle type to train an accurate surrogate model.

**Figure 3:**
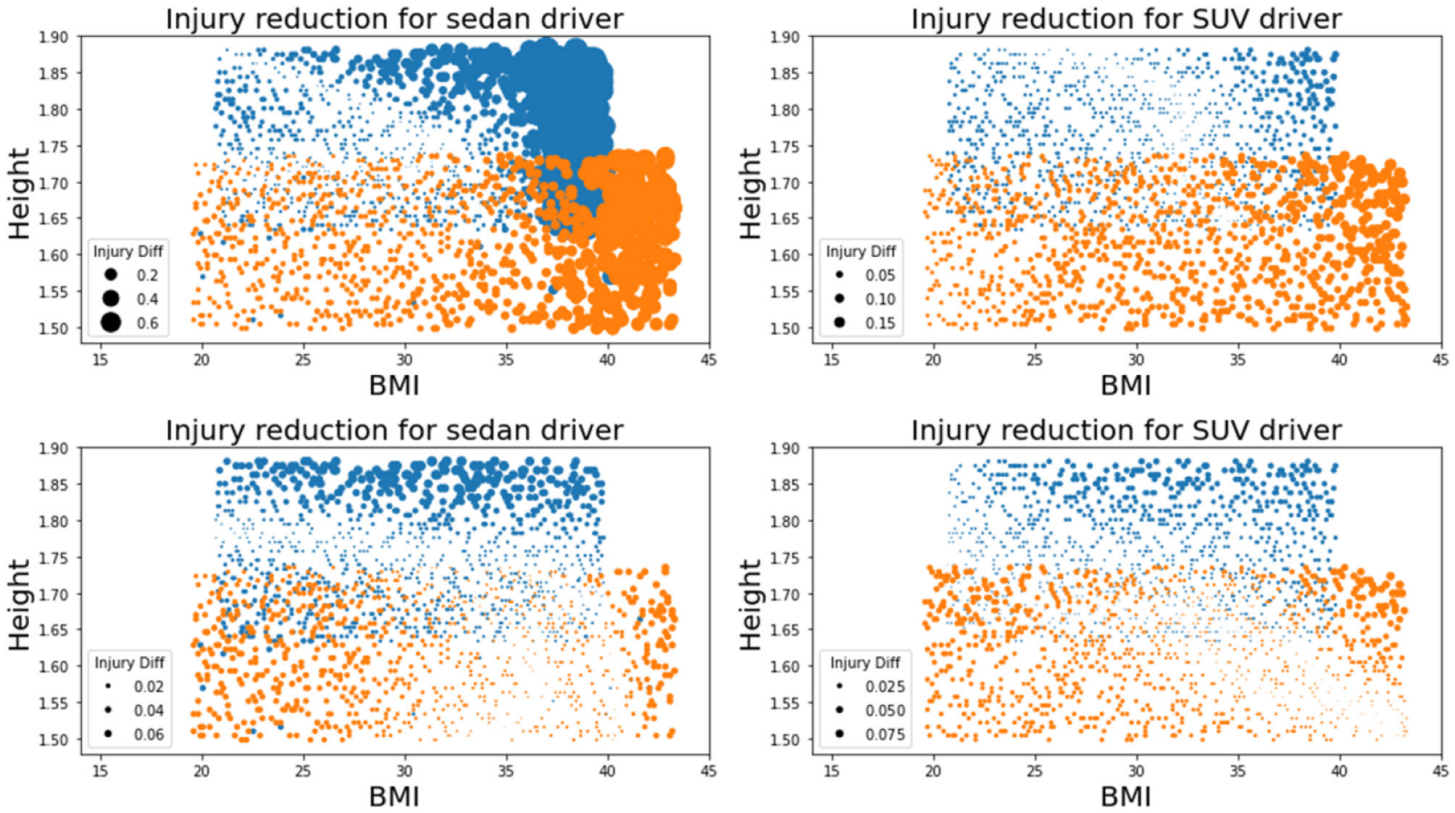
Visualization of injury reduction due to the adaptive design. Blue dots represent males and orange dots represent females. The dot diameter indicates the amount of Pjoint reduction. **(Top left panel)** Reduction in Pjoint of sedan drivers by changing the traditional state-of-the-art design [design (i)] into the adaptive design policy [design (iii)]. **(Top right panel)** Reduction in Pjoint of SUV drivers by changing the traditional state-of-the-art design [design (i)] into the adaptive design policy [design (iii)]. **(Bottom left panel)** Reduction in Pjoint of sedan drivers by changing the state-of-the-art design optimized for the whole population [design (ii)] into the adaptive design policy [design (iii)]. **(Bottom right panel)** Reduction in Pjoint of SUV drivers by changing the state-of-the-art design optimized for the whole population [design (ii)] into the adaptive design policy [design (iii)].

**Figure 4:**
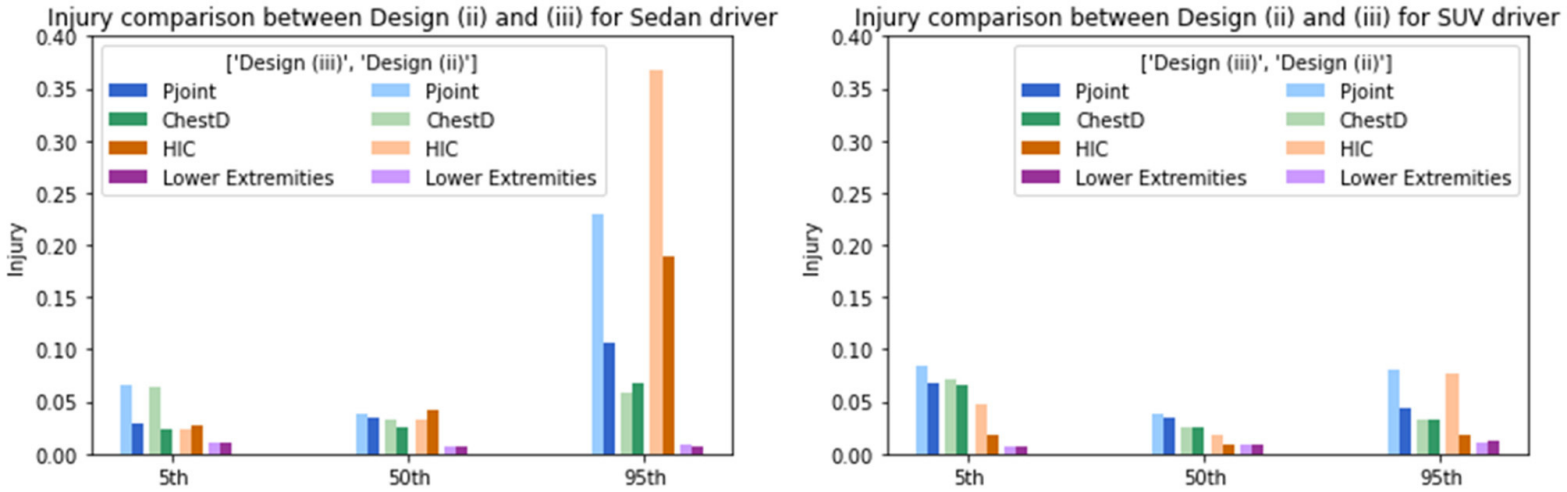
Injury response comparison between design (ii) and optimal design (iii) for the 5th, 50th, and 95th HIII models considering Pjoint, ChestD, HIC, and lower extremities. **(Left panel)** Optimal injury comparison between design (ii) and design (iii) on 5th, 50th, and 95th HIII drivers in SUVs. **(Right panel)** Optimal injury comparison between optimal design for the whole population [design (ii)] into the adaptive design policy [design (iii)] on 5th, 50th, and 95th HIII drivers in sedans.

The proposed adaptive restraint design optimization is then applied to the generated simulation dataset. The design policy was expressed in the polynomial form with the maximal order of three. The probability density function *p*(*s*) was set as constant to assign equal weights to occupants with different anthropometry. The stochastic gradient descent optimizer was used to search for the optimal model parameter for the design policy under a learning rate of 0.001. The effectiveness of the proposed method was tested by comparing the injury risks under (i) the traditional state-of-the-art design optimized for the midsize male, (ii) the state-of-the-art design minimizing the Pjoint among the whole population, and (iii) the adaptive design policy. The comparison results are displayed in Table 2, where columns 2 through 5 show the population injury risks as an average of the Gaussian process predictions at a space-filling design in the occupant covariates space and the last column shows the Pjoint for midsize males. Overall, the design policy leads to an average of 59.28% relative reduction in the Pjoint for sedans, and an average of 24.23% relative reduction in the Pjoint for SUVs, compared to the state-of-the-art design optimized for midsize males. The design policy shows an advantage over the second non-adaptive design optimized for the population, with 17.40 and 13.84% relative reduction in the Pjoint for sedans and SUVs, respectively, implying that the adaptivity in the design policy helps mitigate the population-wise injury risks. It is also worth noting that the second baseline design leads to a higher head injury risk for SUVs when reducing the Pjoint and may increase the injury risk for midsize male occupants, which may compromise the results in the current regulatory or consumer information crash tests that emphasize the head protection. Subsequently, the Pjoint for the midsize male occupants maintained a 0.035 result for both sedan and SUV while further improving the protection for other occupants. The proposed adaptive design does not provide an injury risk for midsize males as low as under design (i) due to the linear assumption of the adaptive design policy function. A visualization of the reduction in Pjoint for individuals is shown in Figure 3. It is worth noting that the adaptive design results in lower Pjoint for individuals with higher BMIs and heights, while achieving a similar protection performance for individuals whose occupant covariates are in the normal range. The injury reduction results indicate that the proposed adaptive design can better protect high-risk occupants with higher BMIs and heights without degrading the current state-of-the-art vehicle design. Specifically, the commonly used 5th, 50th, and 95th Hybrid III (HIII) dummy models are evaluated to demonstrate the injury reduction using the optimal design policy. As shown in Figure 4, the proposed adaptive design policy significantly reduced the injury responses for the three dummies on both the overall injury Pjoint, and the other three measurements including ChestD, HIC, and lower extremities. Also, with the injury scales and vulnerable body parts varying among different dummy models, the adaptive design showed the capability to reduce the injury responses in all body regions and ultimately reducing the overall Pjoint.

**Table 2:** Comparison of average injury risks for the whole population under different optimized designs.

**Sedan**	**HIC**	**ChestD**	**Lower extremities**	**Pjoint**	**Pjoint (midsize male)**
(i) Optimum (midsize male)	0.0911	0.1233	0.1655	0.2542	0.0257
(ii) Optimum (population)	0.0710	0.1049	0.0101	0.1253	0.0383
(iii) Adaptive design policy	0.0535	0.0941	0.0095	0.1035	0.0351
**SUV**	**HIC**	**ChestD**	**Lower extremities**	**Pjoint**	**Pjoint (midsize male)**
(i) Optimum (midsize male)	0.0146	0.0872	0.0085	0.0978	0.0262
(ii) Optimum (population)	0.0254	0.0738	0.0091	0.0860	0.0384
(iii) Adaptive design policy	0.0078	0.0700	0.0088	0.0741	0.0347

**Figure 5:**
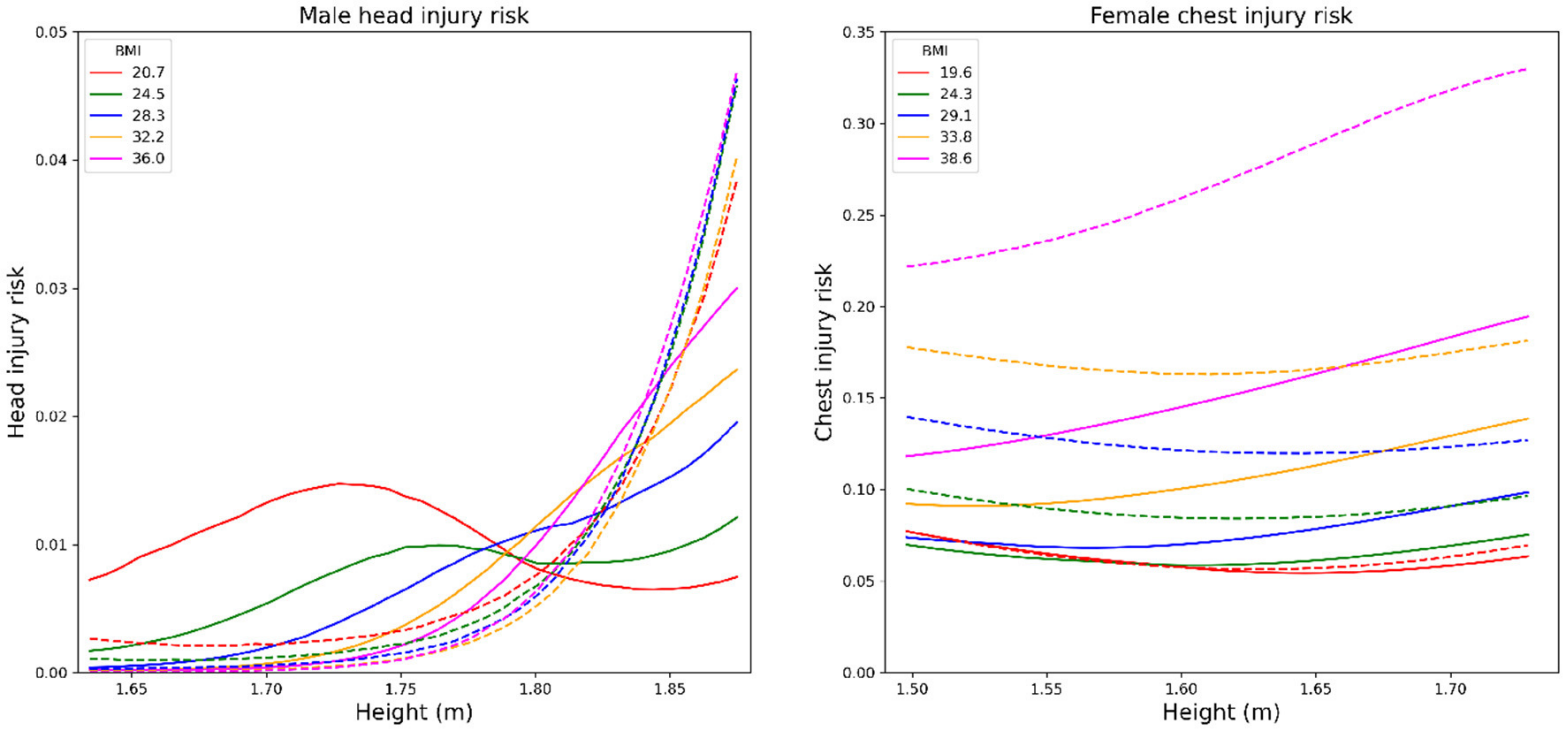
**(Left panel)** Comparison of head injury risks on male between the design policy and the state-of-the-art design for midsize male drivers in SUVs. **(Right panel)** Comparison of chest injury risks on female between the design policy and the state-of-the-art design for midsize female drivers in SUVs. Different colors represent different BMI groups. The dashed line represents the injury risks under the optimal state-of-the-art design for midsize males while the solid lines represent the injury risks under the design policy.

To illustrate how the design policy mitigates the injury risks for the whole population, occupants from five different BMI groups and two sex groups on SUVs were uniformly sampled. As shown in Figure 5, the head and chest injury risks were plotted by height for different BMI and sex groups. Large injury reduction in HIC for taller males and ChestD for all females was observed after applying the optimal design policy. The results in Figure 5 indicates that the design policy mitigates high injury risks for occupants with higher BMI and height compared to the traditional design, which is more significant for female drivers with a high BMI of 38.6.

**Figure 6:**
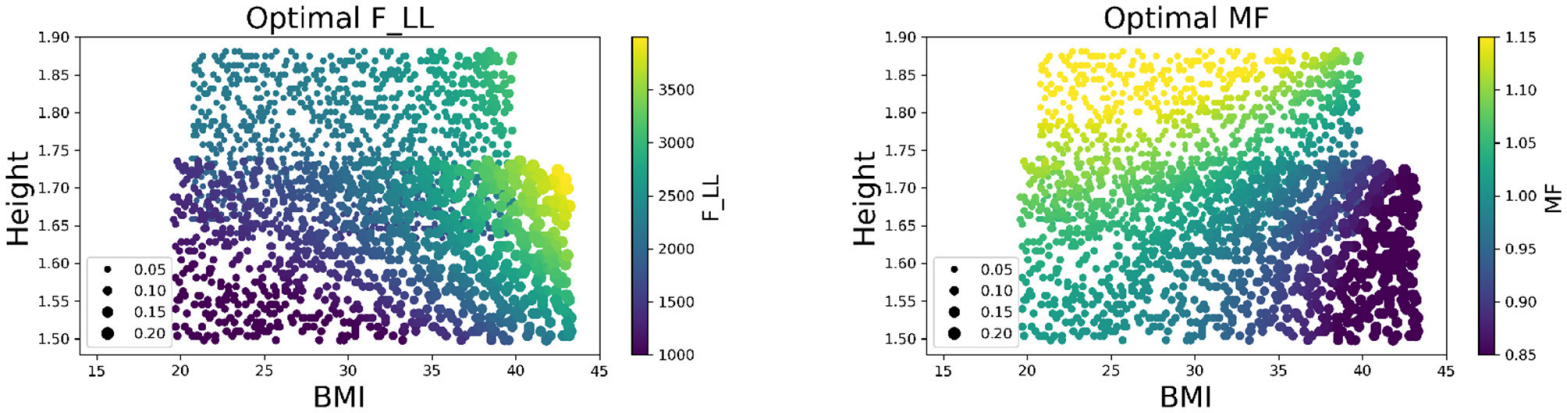
Visualization of design policy for different occupants on SUVs. **(Left panel)** Heat map of optimal F_LL for different occupants. **(Right panel)** Heat map of optimal MF for different occupants. The point size represents the corresponding joint injury risk. The color depicts the value of the design variables.

The trend of the design policy on SUVs is further visualized and validated based on its physical interpretation. Figure 6 illustrates the values of the two vehicle design variables at different configurations of occupant covariates according to the optimal design policy, where the upper section of each plot represents the design policy for males and the lower section represents the design policy for females. An increasing trend of the joint injury risk (depicted by the point sizes) as BMI and height increases for each sex group was observed. The increasing trend was relatively smooth and slow under the proposed design policy, implying that the design policy achieves the balance in injury risks among the whole population. Moreover, the design policy suggests higher retractor belt forces and lower mass flow rates for obese occupants, which corresponds with the vehicle design intuition that a tighter belt force and softer airbag can better protect obese occupants.

**Figure 7:**
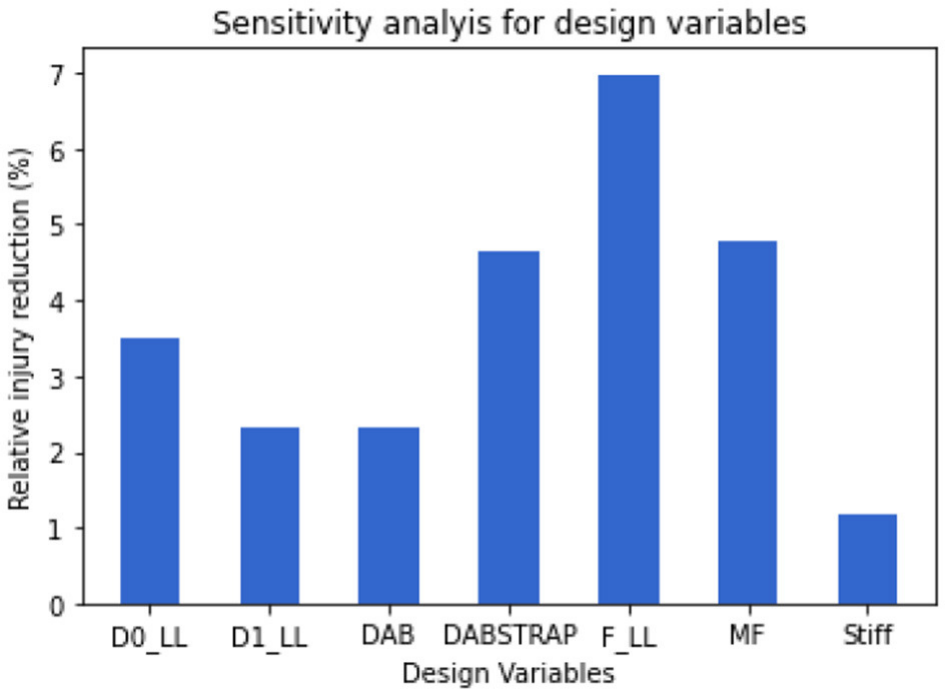
Sensitivity analysis of design variables. The bar plot indicates the relative injury reduction percentage from the second non-adaptive design [design (ii)] to the optimal design by only adapting the current design variable while keeping the other variables the same value as design (ii).

Sensitivity analysis was also conducted to both the surrogate model and the design policy. In the surrogate model, it was observed that the BMI, height, sex, F_LL, and MF were the most sensitive factors influencing the Pjoint. In the optimal design policy, we found the F_LL and MF were sensitive to all three occupant covariates—BMI, height, and sex, especially for boundary conditions where BMI and height increase. The sensitivity of each design variable was quantified by the extent of injury reduction that is only due to the adaptivity of the current design variable, while all other variables take the same value as the second non-adaptive design optimized for the population. Comparing the Pjoint over the population from design (ii), the relative Pjoint percentage reduction with respect to each design variable is shown in Figure 7. Among the seven vehicle design variables, F_LL and MF were able to reduce the population-level Pjoint to a lower level and consequently were the most sensitive design variables with respect to the change of occupant covariates. The general guideline was to use a higher retractor belt force and a lower mass flow rate for obese occupants. Such information may guide the design of the sensing systems for restraint system optimization in future vehicles.

Additional analysis was conducted for the two subgroups of the most vulnerable occupants. Specifically, the group of tall obese males was defined as both BMI and height were larger than the sum of their mean value plus one standard deviation of the male covariates, i.e., BMI is larger than 30.5, and height is larger than 181.7 cm. Similarly, the group of short obese females was defined as the subjects with BMI higher than 37.3 and height lower than 157.0 cm. The optimal design policy showed 25.08% reduction in Pjoint for tall obese males from 0.0854 to 0.0640 and 48.25% reduction in Pjoint for short obese females from 0.2210 to 0.1144.

## Discussion

This study lays out a method to adaptively adjust vehicle restraint systems to further enhance the safety balance. The objective is to tailor the vehicle restraint system for individual occupants through machine learning. The effectiveness of the proposed method was demonstrated by the reduction in population-wise injury risks compared to the traditional state-of-the-art design. The advantage of the conceptual adaptive restraint system lies in the improved protection of the occupants whose subject variables are not considered in the current regulated crash tests. In the meanwhile, the proposed adaptive restraint system achieves a similar protection effectiveness for the majority groups of occupants.

This study has a few limitations. First, the conceptual study only focuses on a frontal crash scenario under limited vehicle types and a fixed crash condition. To generalize the proposed approach, future studies should: (i) extend the method to varying crash parameters including impact speed and impact angle, wherein the vehicle design can be adaptively adjusted based on crash scenarios for injury mitigation. (ii) Analyze injuries in more body regions including the neck and valuation of submarining. (iii) Consider the effects of seat location and sitting posture which could significantly impact the injury risks for the population and potentially enhance the adaptive design policy.

Second, the Madymo parametric occupant model was a rigid body dynamics-based model, which may not fully account for the obesity effects in crash simulations and exhibit differences between the simulated and actual responses. To improve the model accuracy, the state-of-the-art finite element parametric human models (26) coupled with field crash data may be introduced in future studies. A quantitative model for the simulation discrepancy may be developed to calibrate the simulation model.

Finally, this study assumes that many restraint design parameters can be fully adaptive. However, in reality the design adaptability might be limited to fewer design parameters. Nevertheless, the methods developed in this study may provide guidance on which design parameter(s) provides the most benefit in reducing the injury risks for different vulnerable populations. Given the need for further simulations, it would be desirable to iteratively generate simulation runs that can provide more information on the adaptive design to improve the method efficiency. Future studies could explore integrating the simulation generation step with the design optimization step under the guidance of the surrogate model to address this limitation.

## Data availability statement

The raw data supporting the conclusions of this article will be made available by the authors, without undue reservation.

## Author contributions

WS, JL, JH, JJ, KS, RZ, and RM contributed to conception and design of the study. WS, JL, and JH organized the database. WS and JL performed the statistical analysis and wrote sections of the manuscript. WS wrote the first draft of the manuscript. All authors contributed to manuscript revision, read, and approved the submitted version.
