# Supplementary material for: Adaptive restraint design for a diverse population through machine learning
Source: Front Public Health. 2023 Aug 10;11:1202970. doi: 10.3389/fpubh.2023.1202970 (PMC10448517; doi:10.3389/fpubh.2023.1202970)
Supplement: Supplementary file 1 [file Data_Sheet_1.pdf]

Appendix

In this study, MADYMO version 7.7 was used for all simulations. We used MADYMO Scaler v7.7 to scale the baseline Hybrid III dummy models into occupants with different sizes and shapes. They are rigid-body-based models, but the ellipsoid-based geometry and mass/inertia distribution are based on human data from the GEBOD database. Therefore, the mass distribution is more representative to humans rather than the HIII ATDs. The ellipsoid dimensions, joint characteristics, and contact stiffness were all scaled. Similar occupant models have been used in our previous studies (1).

The crash pulses used in the Madymo simulation are displayed in the left panel of Figure 1. The vehicle environment with a Madymo model is illustrated in the right panel of Figure 1. For each simulation the Madymo occupant model was positioned according to a driving posture model developed based on measurements from 68 volunteers (2), in which the occupant covariates, seat height, cushion angle and steering wheel location were used to predict the seat location and occupant hip and eye locations. We believe that the model predicted locations are as realistic as the volunteer data, although variations existed. One of the previous studies (3) shows all the equations for the sitting posture regression model, and an additional Figure 2 [Figure 3 in Hu et al. (4)] has also been attached below to show the occupant locations with varied size and shape.

Figure 3 shows the validation results on 20 male subjects and 25 female subjects uniformly sampled based on covariates. The overall trend of the prediction is consistent with the true Pjoint responses, indicating good predictability of the surrogate model for the optimal design policy. The Root Mean Square Error (RMSE) of mean prediction and upper bound prediction is 0.022 and 0.038. Although the mean prediction error is smaller, the underestimation concern prompts the selection of the upper bound as the prediction. Taking one midsize male (BMI = 25.0, height = 175.0 cm) as an example, the true injury is 0.023, and the mean and upper bound prediction is 0.025 and 0.034 respectively. The prediction error is higher when using the upper bound as the prediction compared to the mean. However, the test obese male subject (BMI = 35.6, height = 183.0 cm) shows that the upper bound gives smaller prediction error with the true Pjoint of 0.061, upper bound of 0.059, and mean of 0.041. When predicting from a surrogate model, injury underestimation is less desirable than overestimation when optimizing the vehicle restraint system. Therefore, selecting the upper bound prediction as a worst-case scenario approach is a more desirable and conservative strategy for estimating injury risks.

The injury reduction for the three injury measurements, ChestD, HIC, and Lower Extremities of SUV and Sedan drivers is illustrated in Figure 4. Note that the adaptive restraint design shows different levels of injury reduction for different body regions with the chest and head injury risk reductions contributing the most to the overall Pjoint reduction. The benefit of the adaptive restraint design is demonstrated significantly among the subjects with boundary covariates which are more vulnerable in the state-of-the-art restraint design.

References

1. Turkovich M, Hu J, van Roosmalen L, Brienza D. Computer simulations of obesity effects on occupant injury in frontal impacts. *Int J Crashworthiness*. (2013) 18:502–15. doi: 10.1080/13588265.2013.809646

2. Reed MP, Manary MA, Flannagan CA, Schneider LW, A. statistical method for predicting automobile driving posture. *Hum Factors*. (2002) 44:557–68. doi: 10.1518/0018720024496917

3. Hu J, Zhang K, Reed MP, Wang JT, Neal M, Lin CH. Frontal crash simulations using parametric human models representing a diverse population. *Traffic Inj Prev*. (2019) 20:S97S105. doi: 10.1080/15389588.2019.1581926

4. Hu J, Zhang K, Fanta A, Jones ML, Reed MP, Neal M, et al. Stature and body shape effects on driver injury risks in frontal crashes: a parametric human modelling study. In: *IRCOBI Conference*. (2017), p. 656–67.

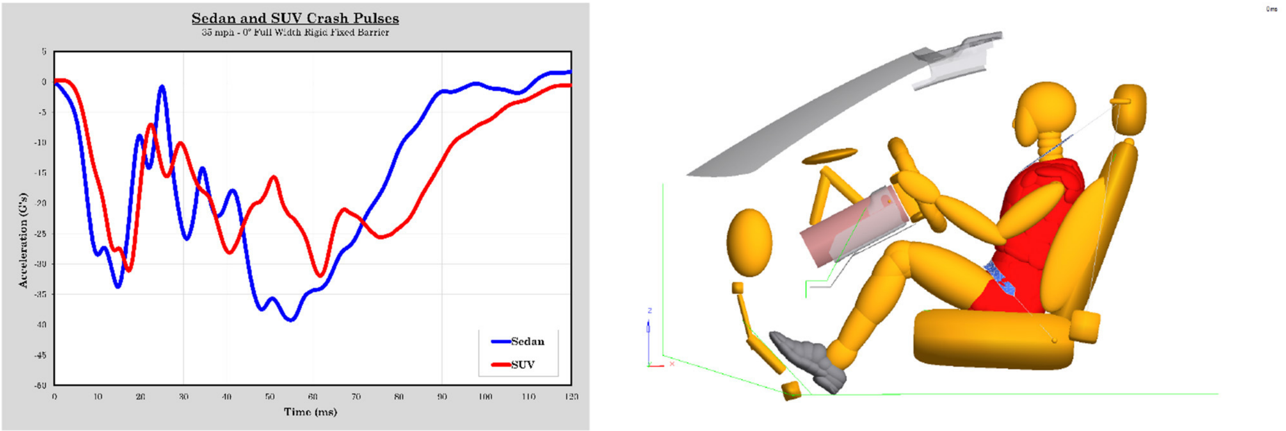

FIGURE 1
Crash pulses and vehicle environment in the Madymo simulation.

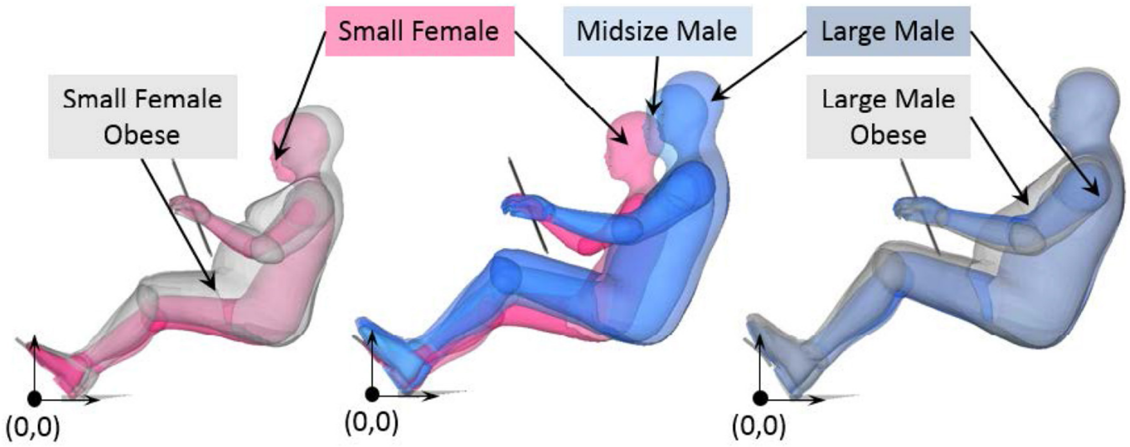

FIGURE 2
Occupant locations with varied size and shape.

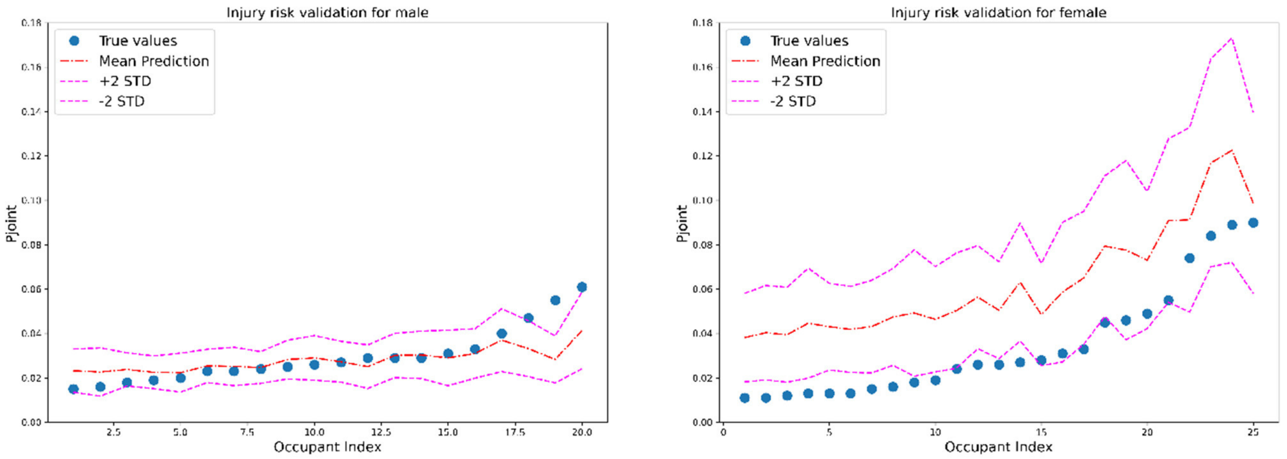

FIGURE 3
Validation results for selected test subjects. **Left panel:** Pjoint results for the 20 test male subjects. The blue dots represent the true Pjoint taking the optimal design policy. The red dashed line is the mean prediction and the purple dashed lines respresent the upper and lower bound of the prediction. **Right panel:** Pjoint results for the 25 test female subjects. The blue dots represent the true Pjoint taking the optimal design policy. The red dashed line is the mean prediction and the purple dashed lines respresent the upper and lower bound of the prediction.

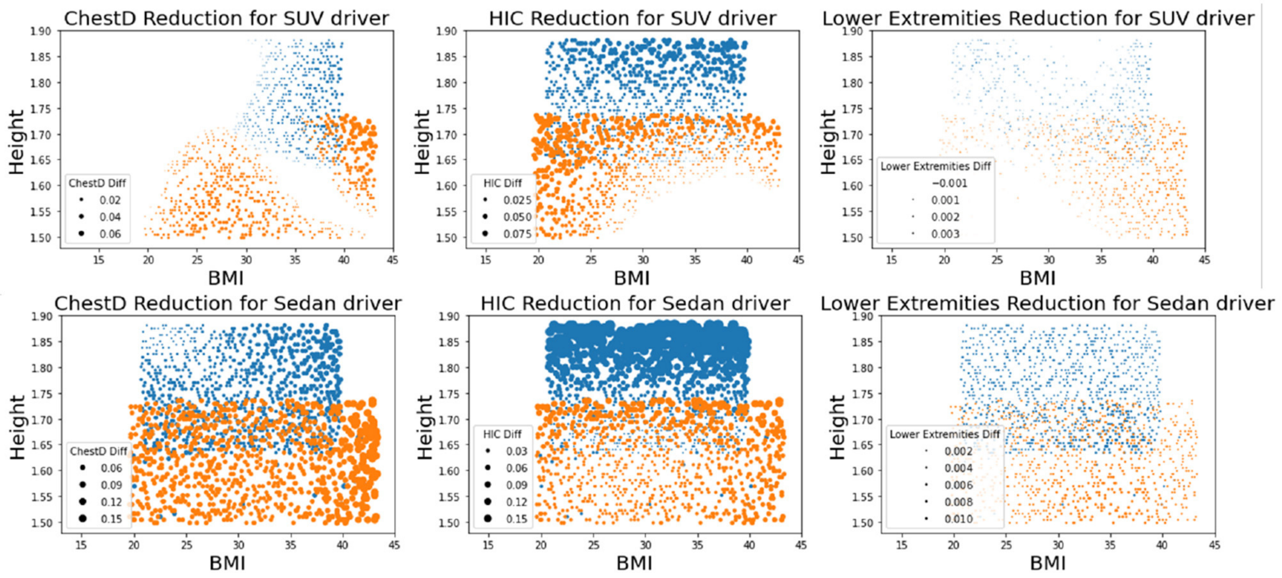

FIGURE 4
Visualization of injury reduction due to the adaptive design. Blue dots represent males and orange dots represent females. The dot diameter indicates the amount of Pjoint reduction. **Top row:** reduction in ChestD, HIC, and lower extremities of SUV drivers by changing the state-of-the-art design optimized for the whole population (design (ii)) into the adaptive design policy (design (iii)). **Bottom row:** reduction in ChestD, HIC, and lower extremities of sedan drivers by changing the state-of-the-art design optimized for the whole population (design (ii)) into the adaptive design policy (design (iii)).
